# Double tendon transfer for massive rotator cuff tear: A case report

**DOI:** 10.1016/j.ijscr.2024.109710

**Published:** 2024-04-28

**Authors:** Roberto Yukio Ikemoto, Vitor La Banca, Thiago Martins Trece Costa, Ana Victoria Palagi Vigano, Joel Murachovsky, Luiz Henrique Oliveira Almeida

**Affiliations:** a Disciplina de Ortopedia - Faculdade de Medicina do ABC, Av. Lauro Gomes, 2000 Santo André, SP, Brazil; b Instituto Brasil de Tecnologias da Saúde (IBTS) R.Visc de Piraja, 407 Rio de Janeiro, RJ, Brazil; c Hospital Ipiranga, Av. Nazaré, 28 São Paulo, SP, Brazil

**Keywords:** Irreparable rotator cuff tear, Tendon transfer, Massive rotator cuff tear, Shoulder, Latissimus dorsi transfer, Lower trapezius transfer, Case report

## Abstract

**Introduction and importance:**

Repairing massive rotator cuff tears (MRCTs) can often be technically challenging due to tendon retraction, bursal fibrosis, and muscular fatty infiltration that usually occurs, often resulting in poor outcomes and an unpredictable prognosis. Although some other surgical management options have been reported, there is a lack of literature supporting tendon transfers in the presence of combined anterior and posterior-superior irreparable rotator cuff tears. We describe a case where a combined transfer of the latissimus dorsi and lower trapezius tendons was employed to treat an MRCT affecting the anterior and posterior superior portions of the rotator cuff.

**Case presentation:**

A 64-year-old male presented significant pain and limited range of motion in the right shoulder following a traumatic anterior shoulder dislocation seven months prior. MRI showed retracted tears (> 5 cm) of the supraspinatus, infraspinatus, and subscapularis tendons with significant fatty infiltration (Goutallier IV). The patient underwent an open transfer of the lower trapezius tendon to the greater tuberosity and the latissimus dorsi to the lesser tuberosity. At the final follow-up, 2.5 years postoperatively, the patient exhibited a painless functional range of motion and could resume daily activities.

**Clinical discussion:**

Although there are alternative surgical options available, the positive outcomes observed in the presented case may be attributed to the restoration of rotational strength and the re-establishment of force coupling across the shoulder.

**Conclusion:**

This report describes the successful implementation of a surgical treatment option for managing MRCT affecting the anterior and posterior superior portions of the rotator cuff.

## Highlights

• Combined transfer of latissimus dorsi and lower trapezius tendons for massive rotator cuff tears
• Non-arthroplasty surgical option for tears involving anterior and posterior-superior regions
• At 2.5 years post-op, patient achieved painless functional range of motion
• Addresses literature gap on tendon transfers for combined irreparable rotator cuff tears

## 1 Introduction

Massive rotator cuff tears (MRCTs) are characterized by multiple parameters, most commonly the number of torn tendons (>2) [1], the total length of the tear (>5 cm) [2], and the percentage of exposed greater tuberosity [3]. While some patients may be asymptomatic, many experience worsening pain and weakness in the affected shoulder, particularly during arm elevation, significantly impacting quality of life affecting daily activities such as washing, dressing, sleeping, housework, food preparation, overhead activities, and work [4].

Treatment options for massive rotator cuff tears (MRCTs) encompass conservative management, debridement, partial repairs, superior capsule reconstruction, subacromial spacer, reverse shoulder arthroplasty, and tendon transfers [5]. Tendon transfers, such as the latissimus dorsi or lower trapezius tendons, have been explored as viable options for posterior-superior MRCTs in non-arthritic, active patients [6,7], as well as latissimus dorsi and pectoralis major tendon transfers for anterior MRCTs [8]. However, successful outcomes hinge on the presence of a functional opposite muscle vector to maintain stable kinematics by restoring rotational strength and force coupling of the shoulder joint. In this case report, we describe a double tendon (combined) transfer procedure with transfer of the latissimus dorsi tendon to the lesser tuberosity and lower trapezius tendon to the greater tuberosity in a patient with combined anterior (subscapularis) and posterior-superior (supraspinatus/infraspinatus) MRCT. Although each tendon transfer (latissimus dorsi and lower trapezius) has been reported individually, their combined use in this context has not been previously documented. This case report followed the SCARE guidelines (Surgical Case Report Guidelines) [9].

## 2 Patient information

A 64-year-old male retired metalworker complained of 7-month pain and strength loss in his right shoulder just after he suffered a shoulder dislocation. On the occasion of the accident, the patient was seen at a hospital where he underwent close reduction and was immobilized with a sling, and he was discharged. Following that, he describes pain that got progressively worse on the anterior and lateral sides of his right shoulder, and he complains of loss of strength, which was limiting his ability to perform daily life activities, as well. Due to COVID-19 pandemic restrictions, the patient did not search for specialized medical care until he was able to. Besides, he said he had no pain in his shoulder prior to the dislocation and no other shoulder injury history. Also, the patient was relatively active, regularly doing home activities and practicing recreational fishing.

## 3 Clinical findings

**Fig. 1:**
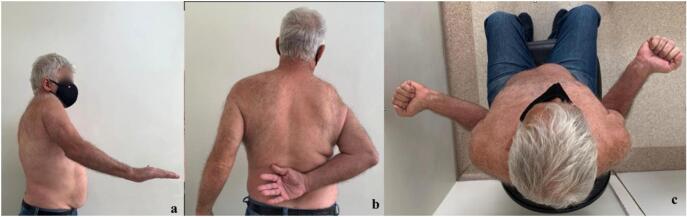
Initial clinic visit active range of motion: (a) Forward flexion of the right shoulder upon initial clinic visit of 31° with noticeable pain. (b) Internal rotation of the right shoulder reaching L1 level, with weakness. (c) external rotation of the right shoulder of 32°.

Upon initial evaluation, the patient presented a limited active range of motion of 31° forward flexion, 32° external rotation, and L1 level internal rotation (Fig. 1a,b,c). Passive range of motion presented no restrictions, with 160° of forward elevation, 50° of external rotation, and T5-level internal rotation. He presented positive Jobe, Patte, and Drop Arm tests and internal rotation weakness (positive Bear Hug and Gerber tests) [10]. His pain level was graded as 5 in VAS (Visual Analog Scale) [11]. No neurological impairment was observed.

## 4 Diagnostic assessment

**Fig. 2:**
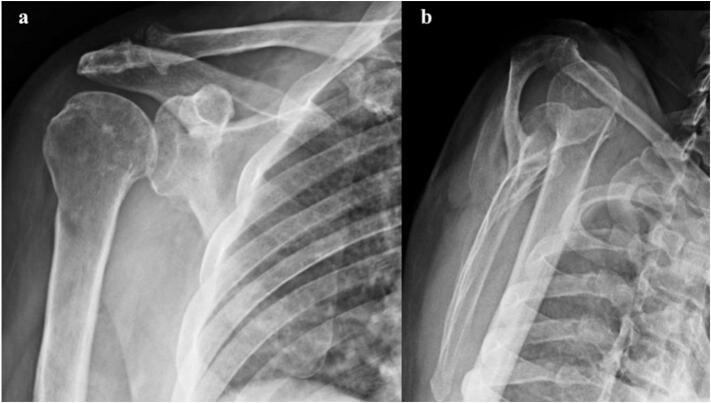
Radiographic study of the right shoulder. (a) AP view of the shoulder, no signs of significant arthritis can be observed; (b) Lateral view, superior and anterior subluxation of the shoulder is noticeable.

**Fig. 3:**
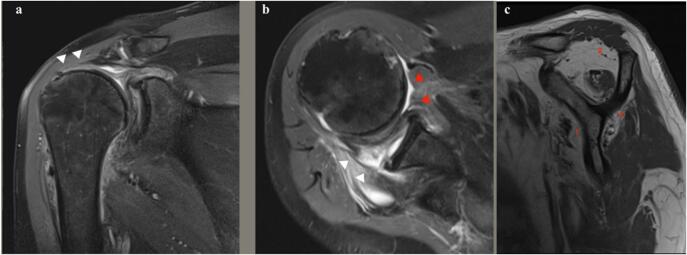
Magnetic resonance imaging of the right shoulder; (a) T2 Coronal view: Tear of the supraspinatus muscle is observed (white arrow) with retraction of the tendon to the level of the glenoid. (b) T2 Axial view: Tear of both the subscapularis tendon (red arrow) and infraspinatus tendon (white arrow) can be observed, with significant retraction of the tendon almost to the level of the glenoid. Anterior subluxation of the shoulder can be observed. (c) T1 Sagital view, significant fatty infiltration of the subscapularis (1), Supraspinatus (2) and Infraspinatus (3) muscles can be observed. (For interpretation of the references to colour in this figure legend, the reader is referred to the web version of this article.)

Plain Radiographies of the right shoulder confirmed the absence of associated fractures, bone defects, or advanced glenohumeral arthritis, but an anterior-superior subluxation of the shoulder was noticed (Fig. 2). Magnetic resonance imaging (MRI) revealed anterior subluxation of the glenohumeral joint with a massive rotator cuff tear with involvement of the supraspinatus, infraspinatus, and subscapularis tendons, which had significant retraction to the level of the glenoid (Fig. 3a,b). Additionally, grade IV fatty infiltration of the supraspinatus and subscapularis muscles (>50 % of the fibers) was observed (Fig. 3c).

## 5 Therapeutic intervention

Considering the severity of the rotator cuff tear and patient impairment in terms of daily activities and range of motion, a decision was reached between the patient and our shoulder group to have the patient undergo a “double tendon transfer” of the latissimus dorsi tendon to the lesser tuberosity and lower trapezius tendon to the greater tuberosity.

**Fig. 4:**
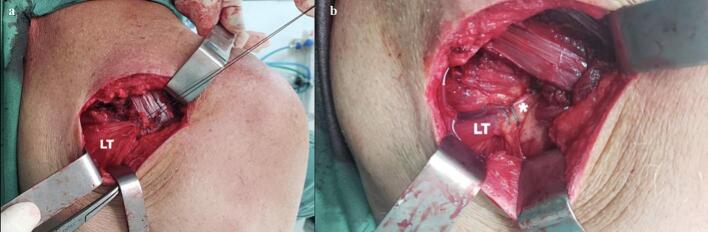
Surgical exposure on the posterior lateral aspect of the shoulder. (a) Lower trapezius tendon (LT) is prepared to be transferred. (b) Lower trapezius muscle is observed reinserted in the greater tuberosity with autograft augmentation (*).

**Fig. 5:**
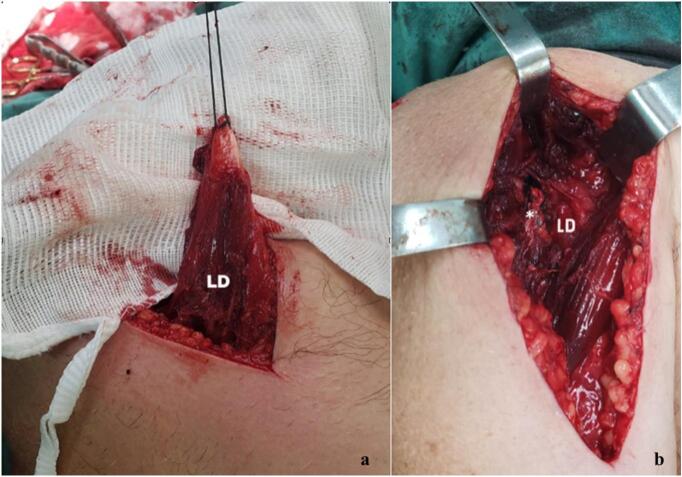
Surgical exposure on the anterior aspect of the shoulder. (a) Latissimus dorsi tendon is isolated and prepared. (b) Latissimus dorsi tendon is transferred to the lesser tuberosity (*).

The surgical procedure was performed with the patient in lateral decubitus position, under general anesthesia and interscalene nerve block. The transfer of the lower trapezius muscle was carried out through an open approach (Fig. 4), following the technique described by Elhassan et al. [12], using a semitendinous autograft fixed to the greater tuberosity with two anchors. Similarly, the latissimus dorsi muscle transfer was conducted using an open technique described by Elhassan et al. [13], with fixation to the lesser tuberosity using anchors (Fig. 5).

Postoperatively, the patient was placed in a shoulder spica brace in 30° of abduction and 50° of external rotation [13].

## 6 Follow up and outcomes

**Fig. 6:**
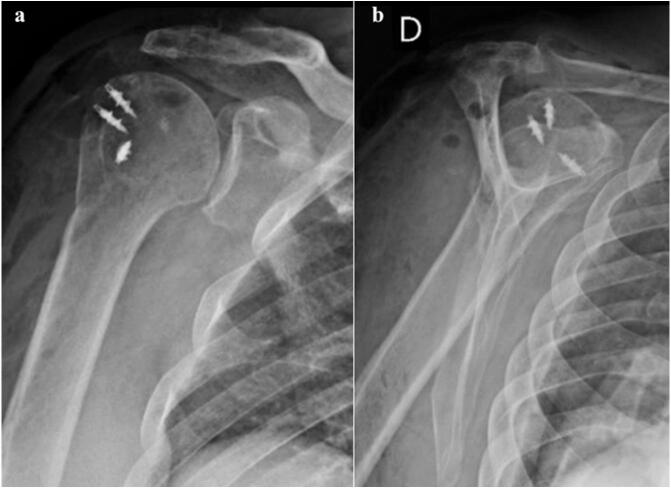
Post operative radiographic study of the right shoulder: AP view (a) and Lateral view (b): No signs of subluxation are identified.

**Fig. 7:**
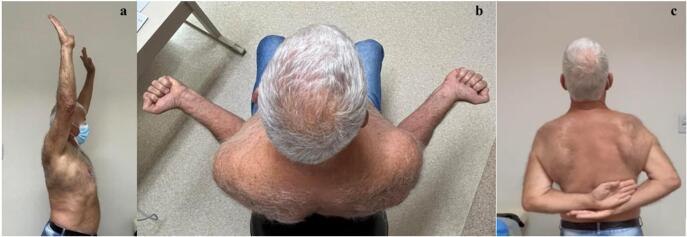
Final follow up active range of motion: Forward flexion reaching 159°, external rotation of 43° and internal rotation at T5 level.

**Fig. 8:**
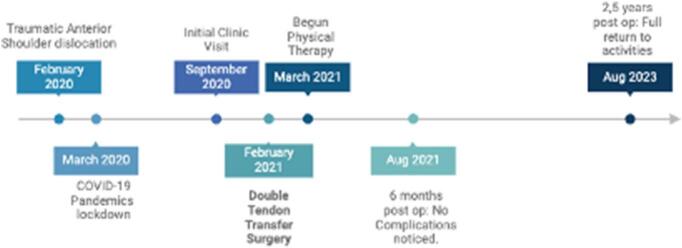
Timeline of relevant events.

No early complications were observed. Post-operative radiographic imaging evidenced no subluxation of the shoulder (Fig. 6). At six weeks postoperatively, he was allowed to begin physical therapy protocol. At the final follow-up two years and six months after the surgery, he had returned to self-reported pre-injury activity levels at home and during leisure time. He did not present any complaints of instability. The active range of motion in the affected shoulder was 159° of forward flexion, 43° of external rotation, and T5 level of internal rotation (Fig. 7), comparable to the contralateral side. His VAS at the final follow-up was graded as 1. A timeline of the events is depicted in Fig. 8 (Fig. 8). Comparison between pre-operative and final follow-up a range of motion and VAS is depicted in Table 1.

**Table 1:** Comparison between pre operative and final follow up active range of motion and VAS.

	Pre Operative	Final Follow-Up (2,5 years)
Active Forward Flexion (°)	31	159
Active External Rotation (°)	32	43
Active Internal Rotation (Level)	L1	T5
VAS	5	1

## 7 Discussion

This report describes a potential surgical approach for managing a relatively uncommon, combined pattern of rotator cuff injury. That case is the first report detailing the combined transfer of the latissimus dorsi and lower trapezius for such a condition, resulting in favorable clinical and functional outcomes.

While the presented case yielded positive results, alternative options should be acknowledged. Isolated transfers of the latissimus dorsi [14] or lower trapezius [15] may be considered in conjunction with the surgical repair of either the subscapularis (if transferred to the greater tuberosity) or supraspinatus/infraspinatus (if transferred to the lesser tuberosity). However, in the presence of MRCTs affecting both, we considered that rotator cuff repair might elevate the risk of failure, potentially compromising tendon transfer functionality. Reverse total shoulder arthroplasty (RTSA) was also considered. However, due to the absence of significant preoperative shoulder arthritis and patient expectations, satisfaction with RTSA was deemed less predictable and consequently ruled out. Like other tendon transfers, certain conditions are considered contraindications for this procedure including shoulder stiffness, glenohumeral arthritis, axillary nerve palsy, and brachial plexus palsy or dysfunction affecting the muscle intended for transfer.

The main strength of this report lies in its innovation. While isolated transfers of the latissimus dorsi and lower trapezius muscles have been previously documented, the combination of both transfers in the same surgical intervention for massive rotator cuff tears (MRCTs), to the extent of our knowledge, has not been reported before. Like any case report, its main weakness stems from the single patient who underwent the technique and the inability to determine its safety and effectiveness in a larger patient population. It's important to note potential complications that can be associated with the described procedure, such as shoulder stiffness, recurrent shoulder instability, infection, and peripheral nerves injury, even though none were observed in the reported case.

We hypothesize that the observed final functional improvement following the double transfer is not solely attributed to the restoration of rotational strength but also the re-establishment of force coupling across the shoulder, thereby partially restoring shoulder kinematics. Compared to other treatment options for MRCTs, this procedure's efficacy extends beyond this study's scope and further research can contribute valuable insights to the existing knowledge on this subject.

## 8 Conclusion

In cases involving MRCTs affecting both the subscapularis tendon and supraspinatus/infraspinatus tendons, with no significant arthritis and active patients, we propose that the double transfer of the latissimus dorsi to the lesser tuberosity and the lower trapezius to the greater tuberosity is a viable consideration.

## Patient perspective

The patient was content with the surgical procedure results as he could return to his daily life and leisure activity, “big-game fishing.”

## Informed consent

Consent for publication was obtained from the patient.

## IRB approval number

CAAE: - Hospital Ipiranga - 75544923.7.0000.5488.

## Ethical approval

Ethical approval for this study was provided by the Ethical Committee of Ipiranga Hospital, São Paulo, Brazil on Dec 20th 2023 under protocol number CAAE 75544923.7.0000.5488.

## Funding

No funding was received for this work.

## Author contribution

Roberto Yukio Ikemoto: Study conceptualization.

Vitor La Banca: Writing the paper.

Thiago Martins Trece Costa: Data Collection.

Ana Victoria Palagi Vigano: Manuscript editing and review.

Joel Murachovsky: Study conceptualization; Manuscript review.

Luiz Henrique de Oliveira Almeida: Project overseeing.

## Guarantor

Roberto Yukio Ikemoto.

## Research registration number

Not Applicable.

## Declaration of competing interest

None.
